# Hantavirus host assemblages and human disease in the Atlantic Forest

**DOI:** 10.1371/journal.pntd.0007655

**Published:** 2019-08-12

**Authors:** Renata L. Muylaert, Ricardo Siqueira Bovendorp, Gilberto Sabino-Santos, Paula R. Prist, Geruza Leal Melo, Camila de Fátima Priante, David A. Wilkinson, Milton Cezar Ribeiro, David T. S. Hayman

**Affiliations:** 1 Departamento de Ecologia, Universidade Estadual Paulista (UNESP), Rio Claro, Brazil; 2 Molecular Epidemiology and Public Health Laboratory, Infectious Disease Research Centre, Hopkirk Research Institute, Massey University, Palmerston North, New Zealand; 3 PPG Ecologia e Conservação da Biodiversidade, LEAC, Universidade Estadual de Santa Cruz, Ilhéus, BA, Brazil; 4 Center for Virology Research, Ribeirão Preto Medical School, University of São Paulo, Ribeirão Preto, SP, Brazil; 5 Department of Laboratory Medicine, University of California San Francisco, San Francisco, California, United States of America; 6 Vitalant Research Institute, San Francisco, California, United States of America; 7 Instituto de Biociências, Departamento de Ecologia, Universidade de São Paulo (USP), São Paulo, SP, Brazil; 8 Programa de Pós-Graduação em Biodiversidade Animal, Universidade Federal de Santa Maria, Santa Maria, RS, Brazil; University of Tennessee Health Science Center College of Medicine Memphis, UNITED STATES

## Abstract

Several viruses from the genus *Orthohantavirus* are known to cause lethal disease in humans. Sigmodontinae rodents are the main hosts responsible for hantavirus transmission in the tropical forests, savannas, and wetlands of South America. These rodents can shed different hantaviruses, such as the lethal and emerging *Araraquara orthohantavirus*. Factors that drive variation in host populations may influence hantavirus transmission dynamics within and between populations. Landscape structure, and particularly areas with a predominance of agricultural land and forest remnants, is expected to influence the proportion of hantavirus rodent hosts in the Atlantic Forest rodent community. Here, we tested this using 283 Atlantic Forest rodent capture records and geographically weighted models that allow us to test if predictors vary spatially. We also assessed the correspondence between proportions of hantavirus hosts in rodent communities and a human vulnerability to hantavirus infection index across the entire Atlantic Forest biome. We found that hantavirus host proportions were more positively influenced by landscape diversity than by a particular habitat or agricultural matrix type. Local small mammal diversity also positively influenced known pathogenic hantavirus host proportions, indicating that a plasticity to habitat quality may be more important for these hosts than competition with native forest dwelling species. We found a consistent positive effect of sugarcane and tree plantation on the proportion of rodent hosts, whereas defaunation intensity did not correlate with the proportion of hosts of potentially pathogenic hantavirus genotypes in the community, indicating that non-defaunated areas can also be hotspots for hantavirus disease outbreaks. The spatial match between host hotspots and human disease vulnerability was 17%, while coldspots matched 20%. Overall, we discovered strong spatial and land use change influences on hantavirus hosts at the landscape level across the Atlantic Forest. Our findings suggest disease surveillance must be reinforced in the southern and southeastern regions of the biome where the highest predicted hantavirus host proportion and levels of vulnerability spatially match. Importantly, our analyses suggest there may be more complex rodent community dynamics and interactions with human disease than currently hypothesized.

## Author summary

Hantaviruses cause disease in people, mainly following transmission from wild rodents to people through contact with infected excreta. Wild rodents use different habitats, and many survive even in anthropogenically changed environments, but to an unknown extent. The objective of our study was to understand how these rodents respond to habitat change in the landscape, to biodiversity and to climate. We measured the proportion of pathogenic hantavirus hosts in the rodent community. We then investigated the spatial correspondence between this proportion and a vulnerability to pathogenic hantavirus infection index in humans within the Atlantic Forest. We found 12 well represented species of rodents that can carry at least one hantavirus genotype. Despite high variation in the host proportion data, the peaks of human vulnerability to disease occurs at higher levels of habitat diversity in the landscape, intermediate levels of rainfall, and areas with less than 15 species in the local small mammal community. Our results suggest hantavirus surveillance and prevention measures are needed in the south and south-east regions of Brazil where highest host proportions and levels of vulnerability spatially match.

## Introduction

**Fig 1:**
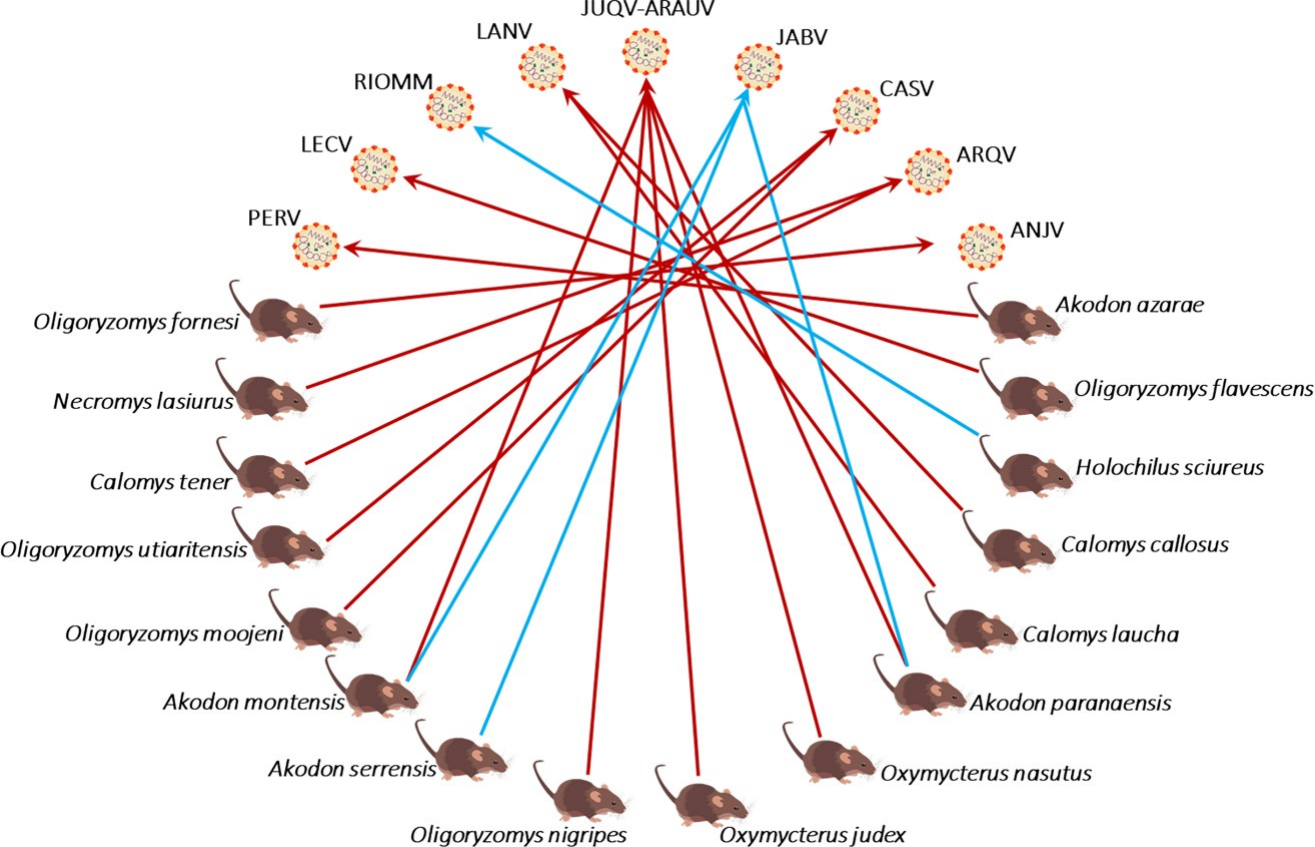
Interactions between hantaviruses and their hosts in South American Atlantic Forest and its boundaries. *Araraquara-Paranoa orthohantavirus* (ARQV), *Castelo dos Sonhos orthohantavirus* (CASV), *Juquitiba-Araucaria orthohantavirus* (JUQV-ARAUV), *Lechiguanas orthohantavirus* (LECV), and *Pergamino orthohantavirus* (PERV) are genotypes of *Andes orthohantavirus* (ANDV); *Rio Mearim orthohantavirus* (RIOMM) and *Anajatuba orthohantavirus* (ANJV) are genotypes of *Rio Mamore orthohantavirus* (RIOMV); *Laguna Negra orthohantavirus* (LANV); *Jabora orthohantavirus* (JABV) [1,15–19]. Known-pathogenic viruses interactions have red connecting lines, others blue.

The members of the genus *Orthohantavirus* (hereafter hantavirus) are three-segmented negative-stranded RNA viruses in the family *Hantaviridae*. More than 90 species of rodents (order Rodentia) and increasing numbers of bats (order Chiroptera) and shrews (family Soricidae) are recognized as hosts, and at least 47 rodent species are known to host pathogenic genotypes of hantavirus [1]. Hantaviruses are spread by host excreta, and it is thought that the main transmission route to humans is via inhalation of viral particles through the respiratory tract, or by direct contact with blood or saliva of infected rodents [2]. Within natural reservoir-hosts, hantaviruses do not cause obvious disease [3], whereas transmission to humans can lead to hantavirus disease with severe cardiopulmonary and renal involvement [4,5]. Hantaviruses are rarely transmitted between humans and human disease is caused by at least 23 recognized viruses carried by specific rodent [6]. In Brazil there are three species of hantavirus causing disease among humans: *Andes orthohantavirus* (ANDV), *Rio mamore orthohantavirus* (RIOMV), and *Laguna Negra orthohantavirus* (LANV) [7]. Within ANDV three genotypes: *Araraquara orthohantavirus* (ARQV), *Juquitiba orthohantavirus* (JUQV), and *Castelo dos Sonhos orthohantavirus* (CASV); within RIOMV the genotype *Anajatuba orthohantavirus* (ANJV) [7–10]. Hantavirus is a putatively emerging virus in Brazil and carried by more than 16 wild rodents which are endemic in the Atlantic Forest and Cerrado biomes [8]. There is a considerable diversity of pathogenic hantavirus genotypes harbored by common wild rodents in the Atlantic Forest of South America (Fig 1). Despite being uncommon, the hantavirus cardiopulmonary syndrome (HCPS) is frequently fatal, with fatality rates reaching 66% among 20 to 34-year old patients, and 45% of infected people in southeastern Brazil [11].

The relationship between biodiversity and pathogen incidence is idiosyncratic [12–14], but host distribution combined with environmental effects are likely the main drivers for hantavirus disease risk in people [1]. Thus, investigation of the relationship between the hosts’ distributions and landscape alteration is of major interest.

Based on a recent framework for understanding drivers of hantavirus disease outbreaks [20], an important component for rodent to human transmission is the proportion of reservoir hosts in a given community. Some ecological correlates of rodent host occurrence suggest land use change, climate, and human disturbance increases host population abundance and thus the proportion of hosts in rodent communities. Furthermore, biotic factors such as the presence of predators and/or competitors and local small mammal diversity can negatively influence reservoir host abundance. In degraded areas, habitat generalist rodents can move through agricultural matrices [21], increasing opportunities for human-rodent contact and viral transmission. In the Atlantic Forest biodiversity hotspot, hantaviruses are mainly associated with the black-footed pygmy rice rat, *Oligoryzomys nigripes* (hosts of JUQV genotype), and the hairy-tailed bolo mouse, *Necromys lasiurus* (host of ARQV genotype). Other sigmodontine rodents (Cricetidae: Sigmodontinae) [22], including at least one more species of grass mouse, *Akodon montensis*, have been found infected with hantavirus and likely play a role in transmission and maintenance of hantavirus genotypes in the Atlantic Forest [15,16,23].

An important landscape attribute that may influence incidence of a pathogen is the arrangement of the contact areas between hosts and humans [24], since this may modulate the intensity by which the hosts use and move through the matrix. Landscape structure can be evaluated in different ways–such as forest cover, edge density, isolation and connectivity–which includes landscape diversity metrics, representing the combination and juxtaposition of different land cover classes as a proxy for habitat diversity, such as forest and agricultural areas. The response of fauna to spatial heterogeneity is still not clear, however a positive association between the suitability for generalist species of rodents and landscape diversity is reported [25,26]. Rodent reservoir hosts of hantavirus seem to be negatively influenced by native forest cover (%), such as *N*. *lasiurus*, which is expected to thrive on agricultural land [27]. Intermediate levels of landscape diversity may reflect a combination of different habitats that can provide suitable areas for nesting and feeding, mainly for species that are not strictly forest-dwelling. For instance, rodents can nest in sugarcane and *Eucalyptus* plantations, where hantavirus disease transmission risk may increase for people that work or pass through these locations.

Here we investigate how potential hantavirus hosts within their mammal communities respond to landscape structure, climate and biodiversity patterns. We test four main hypotheses for explaining hantavirus host proportions: 1) there is a positive correlation with the amount of agricultural matrix; 2) there is a positive relationship with the total habitat diversity of the landscape; 3) that the absence of predators and large competitors (defaunation intensity [28]) and 4) local small mammal diversity would both increase hantavirus host proportions in rodent communities. We used geographically weighted regressions (GW) models to test if these relationships vary in space (see methods). We also explored the correlation between our modelled predictions of hantavirus host distributions and a vulnerability index of human populations to hantavirus infection based on public health surveillance data.

## Methods

### Study region and hantavirus hosts

We analyzed data from areas where target rodents were captured from a set of 283 inventories located within the Atlantic Forest [29] available in the ATLANTIC series database [30]. The ATLANTIC database is the most comprehensive and reliable data set of assemblages for small mammals in the Atlantic Forest–see https://github.com/LEEClab/Atlantic_series. The data were validated through taxonomic correction of reported species, which is crucial for Cricetidae rodents, because they can be misidentified [31]. We evaluated the distribution of potential hosts across sampled sites and did not run models for extremely rare species (S1 Fig). We included all rodents relevant for carrying hantavirus genotypes in the analyses, even when there was no evidence for human disease transmission in the Atlantic Forest, because absence of evidence of transmission does not mean evidence of absence. We also include the interface with vegetation types adjacent with the Atlantic Forest such as boundaries with Cerrado grasslands and Chaco wetlands.

The recognized reservoir hosts of pathogenic genotypes of hantaviruses that occur in the Atlantic Forest and its boundaries (S1 Fig) are *Necromys lasiurus*, *Oligoryzomys nigripes*, *Calomys callidus* (absent in ATLANTIC database), *Calomys laucha*, *Oligoryzomys moojeni* (absent), and *Oligoryzomys fornesi* [9,17]. Hantaviruses can also infect *Akodon montesis* and *Calomys tener* [18], so we also included these species in models along with: *Oligoryzomys flavescens* [32], which is linked to hantavirus transmission in Argentina [33] and Uruguay; *Oligoryzomys fornesi*, the presumed reservoir of ANJV in Amazon [34], but also captured in Cerrado; *Calomys laucha* and *Calomys callosus*, that are considered reservoirs of pathogenic hantavirus in Paraguay (LANV) [19]; *Akodon paranaensis* [17] and *Akodon cursor* (commonly confounded with other *Akodon* species), important hantavirus hosts in Paraguay [19,35]; *Akodon azarae*, which is infected with PERV in Argentina, but with unknown pathogenicity in Brazil [19]; *Oxymycterus nasutus*, a host for JUQV-ARAUV genotypes [16]; and *A*. *serrensis*, a host of JABV that can occur in high altitude grasslands and in Montane forests [36].

### Mapping procedure

Each landscape was mapped in land use and land cover classes with the most appropriate Google image from the start of sampling in QGIS v. 2.0 [37]. Classes of land use were water, initial or medium growth forest, wetland, old growth forest, Cerrado, soil, soy, pasture, citrus, coffee, maize, sugarcane, other cultured crops, tree plantation (*Eucalyptus*), buildings (rural and urban), cloud and others (such as mining areas). Whenever there was doubt on visual interpretation of classes, we used SatVeg time series of spectral signatures to differentiate agricultural uses (https://www.satveg.cnptia.embrapa.br). The mapping and analyses were performed at a 1-km buffer radius from the centroid of the 283 sampling sites, with the minimum resolution for land use and cover analysis of 5 m in order to guarantee a map scale of 1:10,000. We calculated the area and perimeter of polygons using R [38]. We calculated the amount of each class divided by the area of buffer times 100 –i.e. %–, and edge density as the sum of the polygon’s perimeter for each class divided by landscape area (m/m^2^). Although we could not assess the accuracy of sampling site coordinates, the centroid of each landscape represented the most accurate measure of location coordinates from the dataset.

Landscape heterogeneity was used as a proxy for habitat diversity, and was calculated based on the presumed capacity of target rodent hosts to use resources outside forest fragments, in open agricultural areas, and other native vegetation areas [20]. Shannon diversity index from Vegan package v. 2.4.2 [39] was used to calculate habitat diversity in each landscape. The calculation included the amount of each one of the agricultural and native land cover classes mapped, and were also based on the 1 km buffer radius from the centroid of the 283 sampling sites. This extent has been shown to be relevant in landscapes with varying amounts of habitat for small mammal fauna [40,41]. We did not include patch area effects in our models, since the major predictor of woodland small mammal species is habitat amount [41]. Rainfall and average temperature were extracted from WorldClim’s mean of monthly precipitation (in mm) and average monthly temperature (C°*10) (http://www.worldclim.org/), considering the average for area from each landscape unit.

### Defaunation intensity

We conducted a literature search for studies of medium and large-sized mammals in the same or nearby localities in which we had found the assemblage of small mammals to assess their presence or absence. We also searched for papers from gray literature, and included inventories of medium and large mammals [42]. More information on calculations is provided in Bovendorp et al. [43]. To calculate defaunation intensity for each site, we overlapped extent of occurrence maps from IUCN Red List website [44] for 33 species of medium and large sized mammals belonging to Carnivora (23 species); Artiodactyla (nine species); and Perissodactyla (one species). The large mammal species used here were selected due their potential direct competition with and predation on small mammals. Ungulates can modify small mammal habitats by trampling and competing for food with small mammals while carnivores prey on small mammals [45–47]. This information was used to calculate the defaunation intensity of medium and large bodied mammal for each site in our dataset using the defaunation index [48]. We used the occurrence data to generate an expected medium and large mammal community. We calculated the defaunation index (DI) as follows

$$\mathrm{DI}=1-\frac{\sum_{j}^{N}{\mathrm{BWES}}_{j}}{\sum_{i}^{N}{\mathrm{BWES}}_{i}}$$

where *N* is the total species richness in the community; *j* is the total species richness in the actual community composition; *i* is the total species richness in the potential community composition; *BWES* is the average body mass for each species of medium and large mammals. DI ranged from 0 (no defaunation) to 1 (completely defaunated).

### Collinearity evaluation

We assessed the collinearity of the following predictors through Kendall's correlation (τ) analysis: small mammal local richness, defaunation index, temperature, rainfall, edge density of sugarcane, tree plantation, maize, pasture, and forest, land cover of sugarcane, tree plantation, maize, pasture, old growth forest, and habitat diversity. We considered suitable covariates to have pairwise τ values <±0.4 and with p-values >0.05 (S2 Fig). Rainfall was negatively correlated with temperature (τ = -0.46, p <0.05), and we kept rainfall as a predictor. Land cover of old growth-forest was negatively correlated with habitat diversity (τ = -0.69, p <0.05), so we kept habitat diversity. Regarding agricultural matrices, we kept the amount of pasture, sugarcane, tree plantation and maize. Edge density for most land cover types were correlated with land cover, so we kept land cover as the main predictor and dropped edge density from the set of predictors, as land cover is a more intuitive metric.

### Model selection

After the selection of least correlated predictors, we checked for remaining collinearity by using the variance inflation factor (VIF). We found a small value (1.96) and proceeded with the following variables in the model selection procedure: small mammal local richness, defaunation index, rainfall, land cover of sugarcane, tree plantation, maize, pasture, and habitat diversity. Prior to analysis, we standardized covariates to zero (Z-score).

Spatial pattern can be a crucial determinant for species distribution, and should be incorporated as a potential factor influencing aspects of community composition and within a variety of contexts, especially when numerous sites across a biome are considered. For that reason, we conducted a comparison between models with and without geographically weighted terms. We iterated a forward algorithm for selecting geographically weighted models (GW models, [49,50]) based on our hypotheses via calibrating all the possible bivariate GW models by: 1) sequentially regressing a single predictor against each dependent variable; 2) finding the best performing models (minimum AICc values), and permanently including the corresponding predictor in subsequent models; 3) sequentially introducing a predictor from the remaining group predictors to construct new models with the permanently included independent variables, to determine the next permanently included variable from the best fitting model that has the minimum AICc value; and 4) repeating step 3 until all the variables are permanently included in the model. After that we selected the plausible models from where the algorithm kept the AICc values lowest. Local regressions for all covariates (a complete GW model, when all covariates are non-stationary predictors) might not be always meaningful or add relevant explanation (balanced with model complexity and parsimony). This means that not all factors meaningfully vary in space. When a covariate effect does not spatially vary in models, we call it a global (fixed) covariate, because it will have one slope (β) for the entire regression, no matter the spatial coordinate. Local regression coefficients provide local estimates for each response variable. When part of the model varies in space, we call it a mixed model with local and global covariates. After evaluating the lowest AICc model, we tested the hypothesis of non-stationarity for each model covariate via a Monte Carlo procedure (N = 999). When p-values were <0.05, we consider the term as varying in space (non-stationary) and re-run the mixed geographically weighted model maintaining as fixed the coefficients that were not significantly non-stationary. Thus, the mixed GW model is an intermediate between a global (fixed) model and a complete GW model. This step guaranteed parsimony for final model interpretation and generalization of predicted effects for the proportions. Thus, our final best supported model types were based on Monte Carlo estimated p-values.

### Model calibration

We built models for each target species and category using GW model [49,50]. These models allow us to explore responses influenced by regions, and where the residual variation tends to be less spatially autocorrelated [51]. The GW models generate a formula for every sampling unit of the dataset incorporating the response and local predictors falling within a pre-defined bandwidth with a Gaussian function for the spatial decay in similarity. This can complicate generalization but can also be useful to address uncertainty or dynamic patterns. We defined the optimal distance using a calibration procedure. Since the Atlantic Forest is a large biome with more than 1 million km^2^ [29], but we have sparse and irregular sampling sites, we chose an adaptive bandwidth for each response variable (species and category, S1 Table), which suits irregular sample configurations. Adaptive bandwidths can ensure sufficient local information for each local calibration. During calibration we used Albers equivalent projection with datum SAD69 (EPSG 102033).

### Known hosts, viral potential pathogenicity, and species

We used capture number and hantavirus host proportion in a community as proxies for local abundance of host species, and thus the model error terms and link function distribution were declared linear. Mean correlation between species abundance and their proportion was high, validating the use of proportion as proxies for abundance for each species (Kendall's τ = 0.62, se = 0.06, S1 Table). We calculated the proportion of hosts of potentially pathogenic hantavirus genotypes by summing the capture number of all potential host species dividing this by the total number of captures. We had categories of the proportions of hosts of ARQV, JUQV and LANV through summing their respective hosts values. We also divided all hosts between those for which the viral pathogenicity is known and all hosts, including those for which pathogenicity is unknown.

Since sampling effort is an important attribute for determining the number of captures in rodents [52], we included it in our models as a fixed effect. We could not run different sets of models for the subset of data from pitfall traps, live-traps, and when both were used, since the sample sizes for those methods greatly varied, with 13 sites using pitfalls, 211 used live traps and 59 both. Thus, we ran models for the 283 sites altogether regardless of capture method, but accounting for effort. We did not run models for extremely rare species, since model convergence is limited.

### Reservoir host and vulnerability to hantavirus infection in humans

After evaluating models for each host type, we checked the nexus between predicted host proportion and local vulnerability to hantavirus infection using geostatistics. The local vulnerability to hantavirus infection index was calculated through a multi-criteria analysis using public health surveillance data developed by de Oliveira et al. [11]. This index classified the municipality as low (value = 1) to high (value = 5) in terms of vulnerability, based on weighted known risk factors from the literature: the human disease incidence of HCPS, economic gains from agriculture, municipal human development index and degree of urbanization. We computed values of vulnerability using NNJoin in QGIS v. 2.18. We compared the vulnerability index with generated rodent reservoir-hosts maps to investigate the nexus of rodent peak values and disease risk levels. Similarities between higher quantiles of predicted host proportions and higher quantiles of estimated vulnerability were estimated between layers generated by Getis Ord Gi [53] geostatistics. We generated a hotspot map for the predicted proportion of hosts with potential to carry pathogenic hantavirus genotypes (reservoir hosts) and another hotspot map for the vulnerability to infection. We considered as clusters locations that matched high-high and low-low neighbor values within 95% confidence intervals for local G values. For those analyses we used the R-package “spdep” [54]. To improve visualization, we used a Voronoi polygon transformation of the sampling locations. Different coding styles can be used to minimize topology-induced heterogeneity on heatmaps. For instance, style “W” (leverage on low connected sites), or “C” emphasis on hubs. Here we used style “S”, which balances possible biases from those two [55].

After building the hotspot maps, we tabulated the matching values. Then, we plotted a smoothed graph with the main spatial predictors of host proportion in the community (for pathogenic genotypes) and the vulnerability clustering levels (coldspots, hotspots and neutral spots of vulnerability to hantavirus disease).

Finally, maps were made using QGIS v. 2.18 with the layer sources being 1) Atlantic Forest limits from Muylaert et al. [29] available at https://github.com/LEEClab/ATLANTIC-limits., and: 2) Bazilian states from the Instituto Brasileiro de Geografia e Estatística (IBGE) archive for 2015, available at ftp://geoftp.ibge.gov.br/organizacao_do_territorio/malhas_territoriais/malhas_municipais/municipio_2015/Brasil/BR/.

## Results

**Fig 2:**
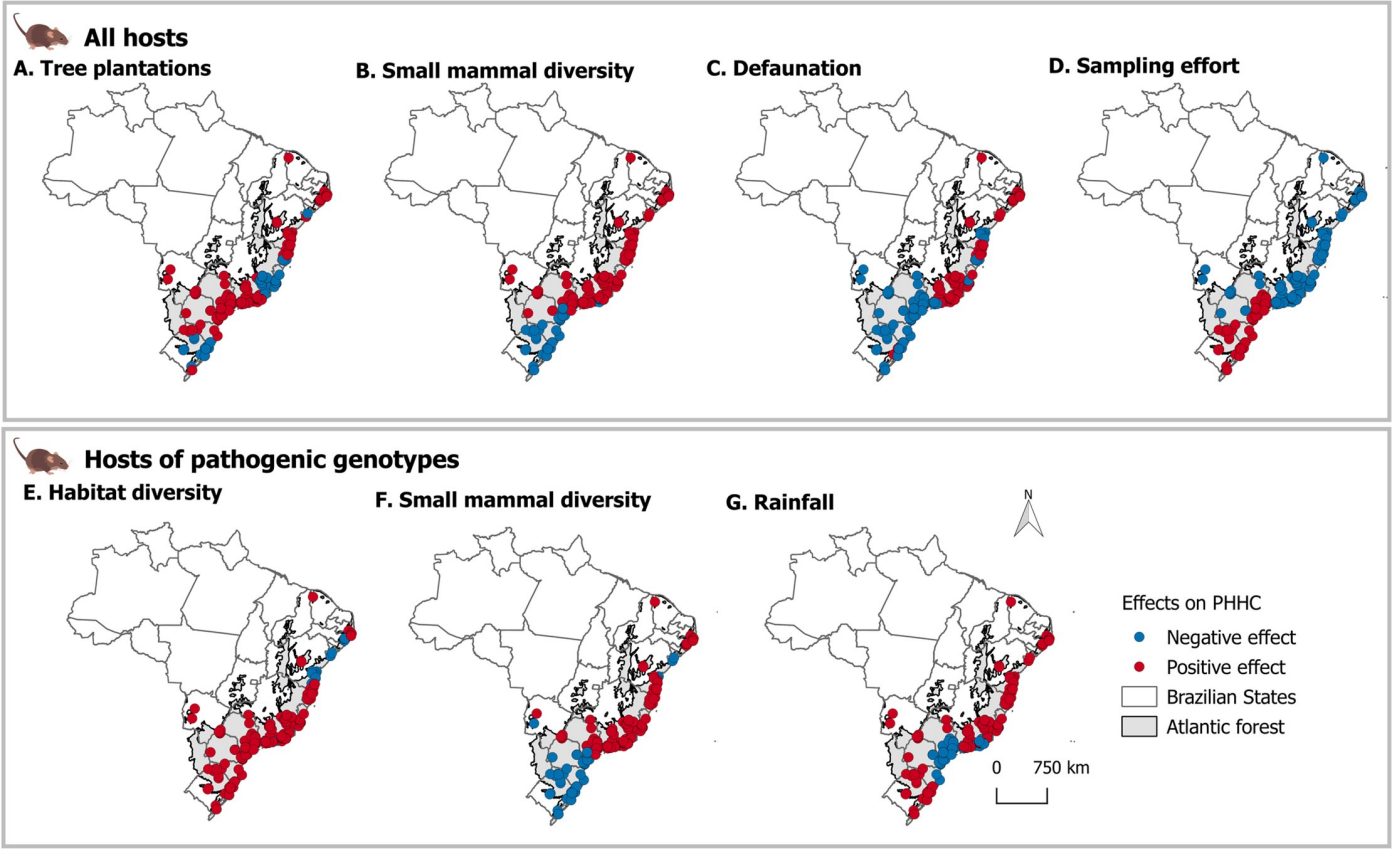
Predicted rodent hantavirus host proportion (PHHC) in the Atlantic Forest rodent community using selected mixed geographically weighted models. A-D: best supported predictors and their spatially varying values for known hosts of hantavirus genotypes (%); E-G: best supported predictors and their spatially varying values for hosts of pathogenic hantavirus genotypes.

The best supported model types for each species and group can be found in Table 1. The summary of the best-supported models and input data can be found in S2 Table. For the group of hosts that can potentiality carry pathogenic hantavirus genotypes the most plausible was a spatially weighted model with an adjusted R^2^ = 0.21. Habitat diversity, rather than habitat type, most strongly influenced the proportion of pathogenic hantavirus hosts in the community. The median slope values for explaining the proportion of pathogenic hantavirus hosts spatially were 7.61 for habitat diversity, 4.08 for rainfall and 1.65 for small mammal diversity. The amount of forestry (β = 0.82) and sugarcane (β = 2.74) in the landscape had fixed positive effects (Fig 2).

**Table 1:** Optimum model covariates for each rodent host group and virus genotype in the Alantic Forest. **Best supported model types for each of the response variables are given, selected based on a Monte Carlo procedure (999 randomizations).** Fixed effects do not vary geographically, and estimates are shown. Geographically varying estimates are provided in S2 Table. R^2^ adjusted values represent the value for a fixed model and a GW model, respectively.

Response variable	Fixed effects	Geographically varying effects	R^2^ adjusted Fixed / GW model
All hantavirus hosts	Habitat diversity (β = 7.67), Sugarcane amount (β = 0.22)	Rainfall, Small mammal species richness, Defaunation index, Sampling effort, Tree plantation amount	0.19 / 0.59
Hosts of pathogenic hantavirus genotypes	Sugarcane amount (β = 0.82), Tree plantation amount (β = 2.74)	Habitat diversity, Rainfall, Small mammal species richness	0.21 / 0.69
ARQV hosts	Sugarcane amount, (β = 2.18), Habitat diversity (β = 2.04)	Rainfall	0.07 / 0.24
JUQV-ARAUV hosts	Habitat diversity (β = 4.14), Maize amount (β = 2.55), Pasture amount (β = 1.95)	Rainfall, Small mammal species richness	0.16 / 0.57
LANV hosts	-	Small mammal species richness	0.06 / 0.03
*Akodon montensis*	Rainfall (β = 2.09), Maize amount (β = 2.75)	Defaunation index	0.16 / 0.42
*Oligoryzomys flavescens*	Tree plantation amount (β = 2.15)	Defaunation index, Pasture amount	0.12 / 0.20
*Akodon cursor*	Habitat diversity (β = 2.08)	Small mammal species richness, Defaunation index	0.02 / 0.49
*Akodon paranaensis*	Rainfall (β = 0.31)	Habitat diversity, Defaunation index, Small mammal species richness, Sampling effort	0.03 / 0.21
*Akodon serrensis*	Habitat diversity (β = -0.36), Defaunation index (β = 0.59)	Rainfall, Sampling effort	0.01 / 0.09
*Akodon azarae*	-	Rainfall, Small mammal species richness, Defaunation index	0.02 / 0.06
*Calomys tener*	Habitat diversity (β = 0.87), Tree plantation amount (β = 1.27), Sugarcane amount (β = 0.43)	Maize amount, Pasture amount, Defaunation index, Average rainfall	0.10 / 0.21
*Oligoryzomys fornesi*	Average rainfall (β = -0.03)	-	0.003 / 0.001
*Oligoryzomys nigripes*	Habitat diversity (β = 2.81), Pasture amount (β = 2.08)	Small mammal species richness	0.01 / 0.32
*Necromys lasiurus*	Sugarcane amount (β = 1.73), Habitat diversity (β = 0.71), Average rainfall (β = 0.51)	-	0.05 / 0.15
*Oxymycterus nasutus*	-	Average rainfall	0.03 / 0.06
*Oxymycterus judex*	Average rainfall (β = 0.23)	-	0.0008 / 0.002

Proportions of JUQV-ARAUV hosts were influenced by average rainfall (β_min_ = -9.41, β_median_ = -13.40, β_max_ = 16.88), small mammal species richness (β_min_ = -14.44, β_median_ = -1.56, β_max_ = 10.65), habitat diversity (β = 4.14), maize (β = 2.55), and pasture (β = 1.95) amounts. Proportions of ARQV hosts were influenced by sugarcane amount (β = 2.18), habitat diversity (β = 2.04), and average rainfall varying spatially (β_min_ = -3.7, β_median_ = 0.71, β_max_ = 3.44). The proportion of LANV hosts in community vary from negative to positive rainfall influence (β_min_ = -0.27, β_median_ = 0.001, β_max_ = 0.03).

Species varied in response to environmental and biotic preditors. *Oligoryzomys flavescens*, *O*. *nigripes* and *A*. *montensis* were positively influenced by rainfall and tree plantations. Maize and defaunation intensity also influenced *A*. *montensis* positively. *Necromys lasiurus* was positively influenced by sugarcane and habitat diversity, but was not affected by rainfall. *Akodon* species varied in their responses to predictors and, with the exception of *A*. *azarae*, all were best supported by mixed models. *Akodon montensis* proportions were explained by rainfall, maize, and defaunation. *Akodon cursor* and *A*. *paranaensis* proportions were influenced by defaunation intensity, habitat diversity and small mammal species richness. *Akodon paranaensis* and *A*. *serrensis* were also influenced by sampling effort, mostly negatively along a spatial gradient (β_median_ for *A*. *paranaensis* = -0.006; *A*. *serrensis =* -0.23). Defaunation affected *A*. *cursor*, *A*. *montensis* and *A*. *paranaensis* differently across space, from negative influence to positive influence (S3 Fig).

For *A*. *cursor* we see the same for northern and coastal areas, but a stronger positive effect in southeastern coastal areas, where more intensively defaunated areas tend to have higher predicted proportions of *A*. *cursor* in the community. For *A*. *montensis*, defaunation had a positive effect on their proportion in southeastern areas, and a negative effect in northern and coastal areas. *Akodon paranaensis*, a species restricted to southern areas, seems to be negatively influenced by defaunation intensity in its core range. Rainfall positively influenced *A*. *paranaensis* proportions in the community (β = 0.31).

*Oligoryzomys flavescens* was influenced by tree plantation (β = 2.15), pasture amount (β_median_ = 0.04, β_max_ = 5.58) and defaunation intensity (β_median_ = -0.21, β_max_ = 0.23). *Oligoryzomys nigripes* was influenced mostly by small mammal species richness, pasture amount and habitat diversity. *Calomys tener* was influenced by habitat diversity (0.87), tree plantation (1.27), sugarcane (0.43), maize (β_min_-1.45, β_median_ = -0.41, β_max_ = -0.22), and pasture (β_min_ = -2.48, β_median_ = -0.45, β_max_ = 0.06) amounts, defaunation intensity (β_min_ = -2.79, β_median_ = -0.38, β_max_ = -0.02), and average rainfall (β_min_ = -2.36, β_median_ = 0.36, β_max_ = 1.25). *Oxymycterus nasutus* (linked to JUQV-ARAUV genotype), *A*. *azarae* (linked to PERV genotype) and the group of LANV hosts (*C*. *laucha* and *C*. *callosus*) were best explained by GW models.

When evaluating all hosts together, regardless of virus genotype they might host, habitat diversity, average rainfall, and small mammal species richness are the best predictors, varying spatially. The best-supported model for all hosts was a semi-parametric model, where the fixed effect was sugarcane, which positively influences host proportion (β = 0.22). Sampling effort consistently had a negative influence on hantavirus host proportion, emphasizing the nature of these rodents being common in degraded areas and being easily captured (even with little capturing effort) throughout the Atlantic Forest. The exception was in southern areas, where higher capturing effort led to the highest predicted proportions of hosts in the community (β_min_ = -6.79, β_mean_ = -3.40, β_max_ = 20.39).

Rainfall was an important predictor for *O*. *nasutus* and *A*. *azarae* (always positive, but varying in space), and small mammal species richness was the main predictor of these species. Defaunation had a negative influence on *A*. *azarae* proportions (β_min_ = -0.25, β_median_ = -0.11, β_max_ = 0.06). We could not fit models for *C*. *laucha*, *C*. *callosus*, *O*. *fornesi*, and *H*. *sciureus* separately because of the scarcity of capture data (less than five observations). *Necromys lasiurus* and *O*. *judex* proportions were best explained by fixed models, but with low adjusted R^2^ of respectively 0.05 in fixed effect model versus 0.15 in GW model, and 0.008/0.002. Despite increased adjusted R^2^ for the GW models, the observed spatial variation might be due to random noise and the low number of observations of this species (S1 Fig, and see estimated p-values at S2 Table). *Akodon montensis*, *C*. *tener*, *O*. *nasutus*, *O*. *judex*, and *O*. *nigripes* can be found in native vegetation areas but also on abandoned pastures and *Eucalyptus* forests [48]. *Necromys lasiurus* populations are positively influenced by habitat heterogeneity and a weak but positive effect of rainfall (β = 0.51) and sugarcane (β = 1.73). *Necromys lasiurus* proportions in the community peaked at intermediate to high levels of habitat heterogeneity at landscape scale. Local rodent diversity was not associated with *N*. *lasiurus* proportions.

### Reservoir host and vulnerability to hantavirus infection in humans

**Fig 3:**
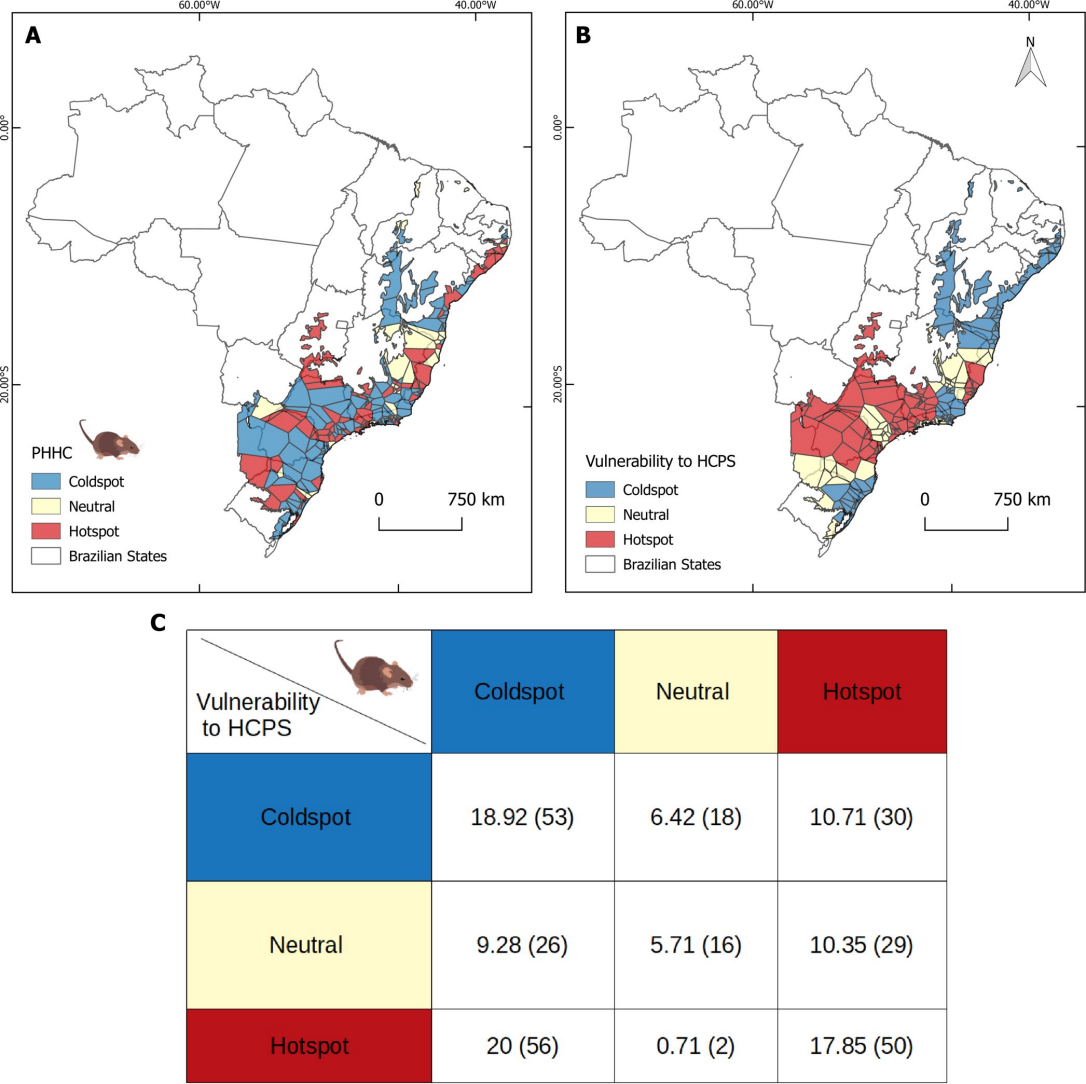
Hotspot maps. **A. Predicted pathogenic genotype hantavirus host proportions in the community (PHHC). B. Human vulnerability to hantavirus disease (HCPS) of municipalities within the Atlantic Forest.** For comparison we used Voronoi polygon optimization based on coordinates of 280 sampling sites. **C.** Spatial matching (%) of hotspots and coldspots of both variables. Number of Voronoi polygons in each class is in parentheses.

We found significant clustering for low and high values of hantavirus host proportions and for the vulnerability index for hantavirus infection leading to HCPS (Fig 3). There was a 17% match between both hotspot maps, and 20% of non-correspondent coldspots and hotspots between maps. Both maps had similar total numbers of hotspot sites (N_Vulnerability_ = 109, N_Hosts_ = 110), but the host map had a higher number of neutral sites (N_Vulnerability_ = 37, N_Hosts_ = 71).

**Fig 4:**
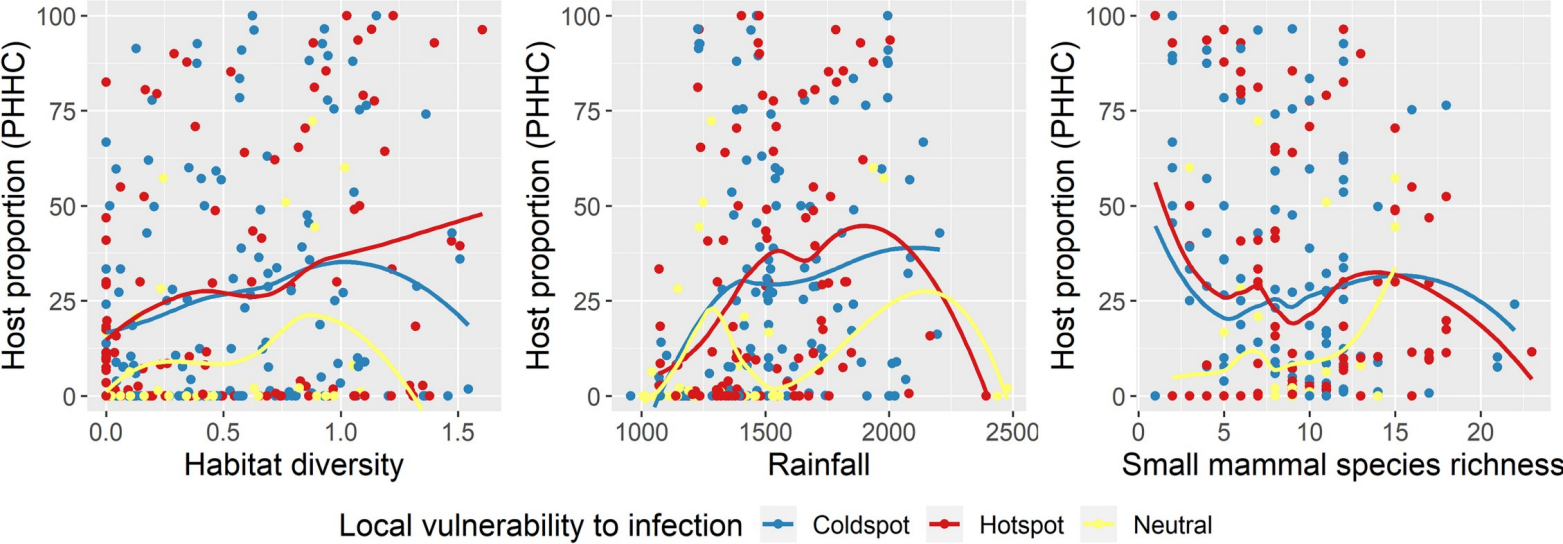
Best supported predictors from geographically weighted models explaining hosts of potentially pathogenic hantavirus genotypes proportions in rodent communities (PHHC) in the Atlantic Forest. The colored loess lines indicate the level of spatial clustering of vulnerability to hantavirus infection in humans.

When we visualize the direct effects of habitat diversity, rainfall and small mammal richness on host proportion there is overdispersion (Fig 4), likely because the effect of predictors varies along the environmental and spatial gradients. However, it enables us to explore how host proportions change at different vulnerability clustering levels (hotspots, coldspots and neutral spots). In general, for hotspots the host proportion in community increased with habitat diversity and was higher in areas with intermediate values of rainfall. The highest proportions of hantavirus hosts were found in areas with low to mid-levels of rainfall (between 1500 and 2000 mm). For comparison purposes, the average rainfall in Brazil is 1761 mm (FAO, https://data.worldbank.org/indicator/AG.LND.PRCP.MM). Also, the host proportions in hotspots seem to increase in areas where small mammal richness is below 15 species.

## Discussion

In this study we link biodiversity and hantavirus host proportions in local assemblages across the entire Atlantic Forest biome. Landscape level amounts of sugarcane and tree plantation positively influenced hosts of potentially pathogenic hantavirus genotypes (Hypothesis 1). Our models suggest an overall positive influence of habitat diversity and rainfall on pathogenic host proportions in the community (Hypothesis 2). Moreover, we discovered that the hantavirus host proportions in rodent communities are positively influenced by local species numbers in most of the Atlantic forest (Hypothesis 3), but that local species richness seems to be a diluting force in southern communities. Defaunation intensity had a geographically varying influence for known hantavirus hosts but did not seem to influence the proportion of the hosts for pathogenic viruses in local rodent communities (Hypothesis 4).

A recent model developed for predicting hantavirus case probability in the Atlantic Forest and Cerrado concluded that native forest cover did not predict case probability, but that sugarcane and social development index did [56]. Sugarcane amount was a good predictor of the proportion of hosts of potentially pathogenic hantavirus genotypes, such as *C*. *tener* and *N*. *lasiurus*. In a local study in the state of São Paulo, landscapes with higher amounts of sugarcane plantation had a higher prevalence of rodent hosts [57]. Landscape degradation negatively influences rodent diversity [58], while sugarcane areas tend to have higher species dominance, particularly for habitat generalist species [18]. Anthropogenic landscape matrix use can differ among rodent species [59], where generalist hosts might use sugarcane plantations and other agricultural land types as corridors, for nesting and/or as foraging sites [60], in contrast to forest specialist species [61]. From our results, increased habitat diversity influences hantavirus host proportion non-linearly.

Here we used the proportion of rodents in the community as a measure of rodent density. We lack long-term sampling data for most species within the studied biome, which would allow measurement of both infection dynamics and host demography. However, we assume that a higher host proportion in the community can be a proxy for important areas for hantavirus disease, despite us not having the infection rates from the captured rodents. Although many rodent hosts are common—and this should lead to a balance in host proportions in similar communities across space—rodent prevalence might be very low [62,63], and density can influence the infection dynamics of hantaviruses [64]. However, other components that vary temporally, such as contact rates, density dependent infection transmission, and transmissibility [64,65] were not assessed, limiting our power to infer transmission risk.

Here we show that landscape diversity has a positive influence on potentially pathogenic hantavirus host proportions in the Atlantic Forest rodent community, likely in part due to the increased availability of agricultural areas and other native habitat types, such as Cerrado, but this varies in space. Hosts are present on most of the gradient of habitat diversity, but peak with intermediate levels of rainfall, with a decreasing trend in their proportion where there is higher species richness (Fig 4).

The remaining fauna in degraded landscapes has the key characteristic of being miniaturized. Small rodents are less conspicuous, less hunted, have rapid reproduction cycles or large population sizes. The downsized fauna has the potential of performing important ecological processes, including ecosystem services and disservices, but with rapid change in its quality and quantity. Despite being known as "generalists", hosts of known hantavirus genotypes were influenced by defaunation intensity, with the relationship varying across the Atlantic Forest. *Akodon montensis*, *O*. *flavescens*, *A*. *cursor*, *A*. *paranaensis*, and *C*. *tener* were most influenced. Defaunation intensity positively affected *A*. *serrensis* and mostly positively affected *A*. *cursor*. Thus, the absence of predators and herbivores as competitors is beneficial for some hantavirus hosts. We still do not know to what extent this can also affect viral prevalence. Orrock et al. [66] found that predator richness reduces *Sin Nombre orthohantavirus* (SNV) prevalence among rodents, suggesting that the conservation of predators and biological diversity may have benefits on human health via the control of infectious diseases prevalence in wildlife. Although it is tempting to suggest that in our results the hotspots of hantavirus disease vulnerability in the Atlantic Forest may result from a lack of a complex network of interacting species (including predators), the mismatches of hotspots prevents us from making a definitive conclusion (Figs 3 and 4). The increased presence of small mammals reservoirs would tend to increase viral presence [20,67,68], and thus the risk of disease transmission. As our results show, hantavirus host proportion peaks when agricultural expansion is accentuated (Fig 2A), mostly in southern regions. In the southern region of Brazil, where the presence of rodents in grain crops and storage areas is frequent, hantavirus disease cases are the second highest nationally [69]. Since *N*. *lasiurus* often has high infection rates [57,70] we suggest that increased surveillance may be effective in southeastern areas, where they are present in the highest predicted proportions. Local rodent diversity was not associated with *N*. *lasiurus*, suggesting that landscape alteration is more important than other rodent species dominance effects in determining abundance. Indeed, in southeastern Brazil, it has been observed that *N*. *lasiurus* is the most abundant rodent in non-native grassland crops of *Brachiaria decumbens*, where the original vegetation was Cerrado [57]. A more detailed long-term period of monitoring is required to test those associations in detail [70].

Our study has some additional limitations. First, it was developed with secondary data and has spatial gaps that can lead to uncertainty regarding habitat selection. Low capture efforts were sufficient to detect most of the species in our dataset, but some species are inherently rare in the Atlantic Forest, such as open area specialist rodents which are more common in Central Brazil in a typical savanna-like biome. Surveys were conducted with different sampling efforts, and this was taken into consideration in our models as a fixed variable. Seasonality seems to be an important factor influencing rodent abundance, as well as viral prevalence, and this was not accounted for in our study. For instance, Luis et al. [65] demonstrated that seasonality has a great influence on the abundance of *Peromyscus maniculatus*, the sigmodontine rodent host of SNV in Montana, USA; likewise, the abundance of small wild mammals is highly associated with the dry season in an agricultural system within the Atlantic Forest [71]. Importantly, novel questions arise from our results, especially relating to the coexistence between host species in a certain assemblage. How rodents share space and resources, and whether infection spillover risk reaches a threshold are open questions.

There was a weak correspondence between our model of hantavirus host proportions and observed hantavirus infection rates in people, however our hotspot analysis points towards important zones, such as north of the State of São Paulo and southern interior regions, in which spillover to humans is most likely to occur if no prevention is applied. Since 1993 almost 2,000 hantavirus cases have been reported across most Brazilian states. Santa Catarina State, in southern Brazil presents the second highest incidence of the disease. Southeastern Atlantic Forest is the most vulnerable area within the biome in Brazil. This information helps to inform surveillance and prevention of hantavirus disease risk and contributes to the understanding of potential factors influencing disease transmission risk.

Overall, we found that there is a strong effect of space and land use change influencing hantavirus host assemblages at the landscape level across the entire Atlantic Forest. In the central-north areas of the State of São Paulo, one of the hotspots for hantavirus disease (Fig 3B), the most common hosts are *N*. *lasiurus*, *O*. *nigripes* and *A*. *montensi*s [70]. Rodents with diet plasticity such as *N*. *lasiurus* [69,70] and the ability to adapt to disturbance in native ecosystems can rapidly decrease the distance, and thus increase interactions, between humans and the wildlife hosts. This may have further implications, since it increases the probability of human contact with infected rodent excreta.

Hantavirus disease risk is detected in areas populated more densely by reservoir-hosts, as described here in the areas of hotspots convergence between vulnerability to disease and host proportion in community. However, the presence of hosts acts as a simple proxy for the distribution of their pathogens [72] and our results show that defaunation leads to greater dominance of hantavirus hosts in communities of the northern parts of Atlantic Forest, while small mammal richness is associated to reduced dominance of hosts of pathogenic hantavirus genotypes in southern regions. These findings do not inevitably mean more disease risk for people in northern areas (Fig 4), because the prevalence of infection is not necessarily proportional to the community structure. Thus, we should further evaluate infection dynamics in maintenance communities. For example, one study identified a high abundance and broad distribution of suitable hosts with no detection of infection in Rio de Janeiro [63]. Investigating the effects of other biotic factors on virus prevalence across different host populations would also help us to understand whether hantavirus pathogenic genotypes are subject to a dilution effect [64]. Importantly, if there is a dilution effect, then conservation efforts will work synergistically to maintain biodiversity and reduce disease prevalence.

## Supporting information

S1 Table Correlation between the proportion of hantavirus host in a community (PHHC) and number of captures. All correlations were significant (p <0.05) and positive and thus we used proportion in community as the response variable. The adaptive bandwidth used for geographically weighted models and semi parametric models (mixed) are shown. Adaptive bandwidths are the number of neighbors included for local spatial regressions.
(PDF)

S2 Table Input data, best supported models and their statistical descriptors. (XLS)

S1 Fig Capture distribution of the main potential hosts of hantavirus within Atlantic Forest sites. Number of captures are in y axes; dashed lines show the mean value of captures and at the bottom how many sites the species was present.
(TIF)

S2 Fig Model predictors selection based on Kendall’s τ using observational data from Atlantic Small mammal dataset. Crosses indicates non-significant correlations (p<0.05). We only used predictors with correlations lower than 0.4 in magnitude in the same model. S = small mammal local species richness, prec_mean = average rainfall, t_mean = average temperature.
(TIF)

S3 Fig Geographically weighted effect of defaunation intensity of medium and large sized mammals on known geographic ranges of extant *Akodon* species proportion in the rodent community within Atlantic Forest. A. *Akodon cursor*, B. *Akodon montensis* and C. *Akodon paranaensis* proportions as a function of defaunation. Ranges were downloaded from IUCN red list website [44].
(TIF)
