# Supplementary material for: Hantavirus host assemblages and human disease in the Atlantic Forest
Source: PLoS Negl Trop Dis. 2019 Aug 12;13(8):e0007655. doi: 10.1371/journal.pntd.0007655 (PMC6748440; doi:10.1371/journal.pntd.0007655)
Supplement: S1 Table — All correlations were significant (p <0.05) and positive and thus we used proportion in community as the response variable. The adaptive bandwidth used for geographically weighted models and semi parametric models (mixed) are shown. Adaptive bandwidths are the number of neighbors included for local spatial regressions. (PDF) [file pntd.0007655.s001.pdf]

S1 Table. Correlation between the proportion of hantavirus host in a community (PHHC) and number of captures. All correlations were significant ($p < 0.05$) and positive and thus we used proportion in community as the response variable. The adaptive bandwidth used for geographically weighted models and semi parametric models (mixed) are shown Adaptive bandwidths are the number of neighbors included for local spatial regressions.

**Response variable (PHHC)**	**Kendall's correlation**	**Optimum adaptive bandwidth**
Assemblage of Hosts of potentially pathogenic hantavirus genotypes: *Necromys lasiurus, Oligoryzomys nigripes, Akodon montensis, Calomys tener, Oligoryzomys fornesi, Oxymycterus nasutus, Oxymycterus judex, Calomys callosus, Calomys laucha, Oligoryzomys flavescens*	0.42	25
Assemblage of All Hosts: *Necromys lasiurus, Oligoryzomys nigripes, Akodon montensis, Calomys tener, Akodon paranaensis, Akodon cursor, Akodon serrensis, Oligoryzomys fornesi, Oxymycterus nasutus, Oxymycterus judex,Holochilus sciureus, Calomys callosus, Calomys laucha, Akodon azarae, Oligoryzomys flavescens*	0.37	27
Assemblage of Hosts of AQAV: *Necromys lasiurus, Calomys tener*	0.58	52
Assemblage of Hosts of JUQV ARAUV: *Oligoryzomys nigripes, Akodon montensis, Oxymycterus nasutus, Oxymycterus judex, Akodon paranaensis*	0.29	25
Assemblage of Hosts of LANV: *Calomys laucha, Calomys callosus*	0.46	182
Akodon azarae	0.76	182
Akodon cursor	0.33	27
Akodon montensis	0.51	25
Akodon paranaensis	0.64	39
Akodon serrensis	0.6	55
Calomys tener	0.5	53
Necromys lasiurus	0.45	66
Oligoryzomys flavescens	0.79	53

Oligoryzomys nigripes	0.27	39
Oxymycterus judex	0.41	281
Oxymycterus nasutus	0.8	182
