# Supplementary figures and images for: Hantavirus host assemblages and human disease in the Atlantic Forest
Source: PLoS Negl Trop Dis. 2019 Aug 12;13(8):e0007655. doi: 10.1371/journal.pntd.0007655 (PMC6748440; doi:10.1371/journal.pntd.0007655)

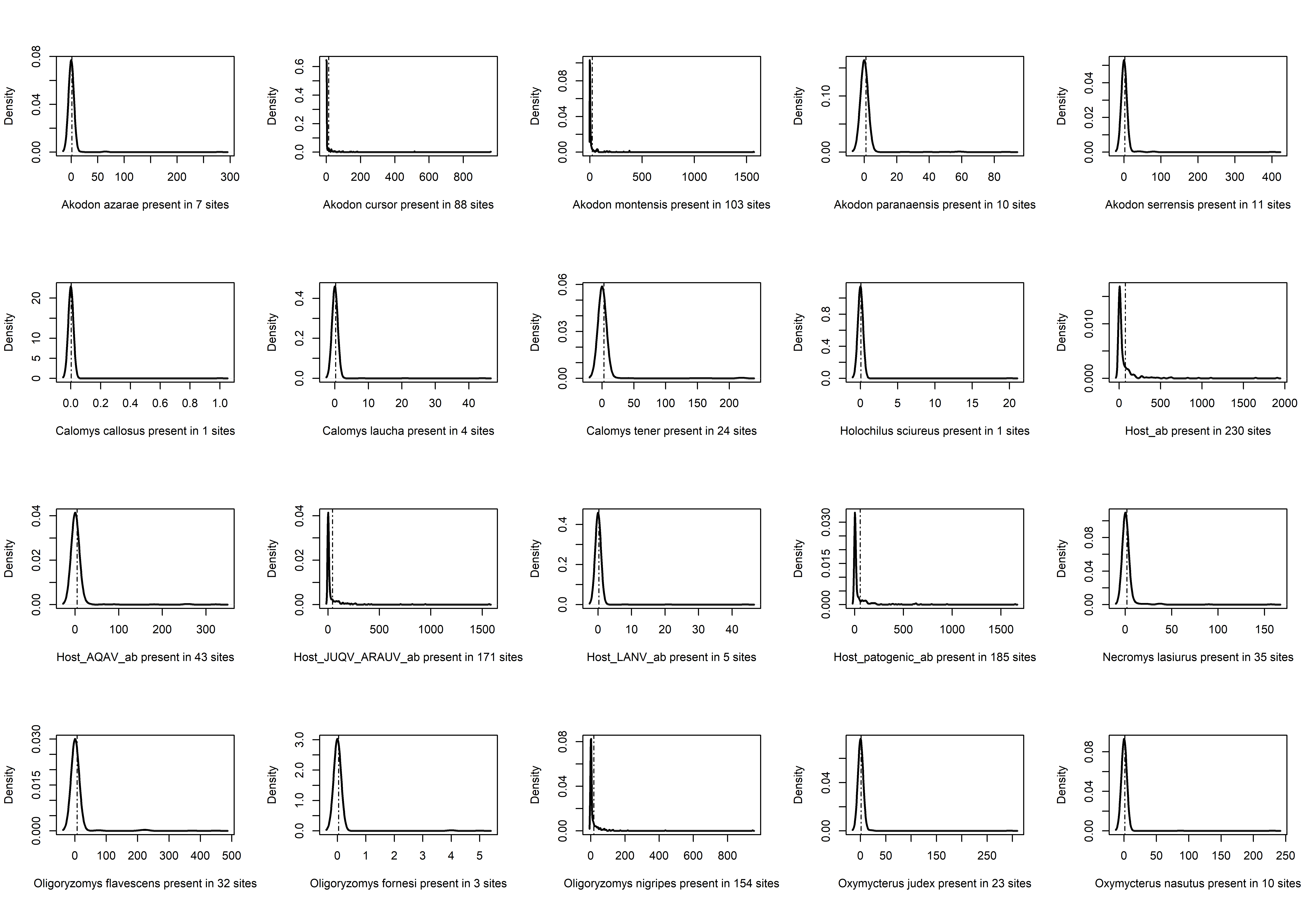

Supplement: S1 Fig — Number of captures are in y axes; dashed lines show the mean value of captures and at the bottom how many sites the species was present. (TIF) [file pntd.0007655.s003.tif]

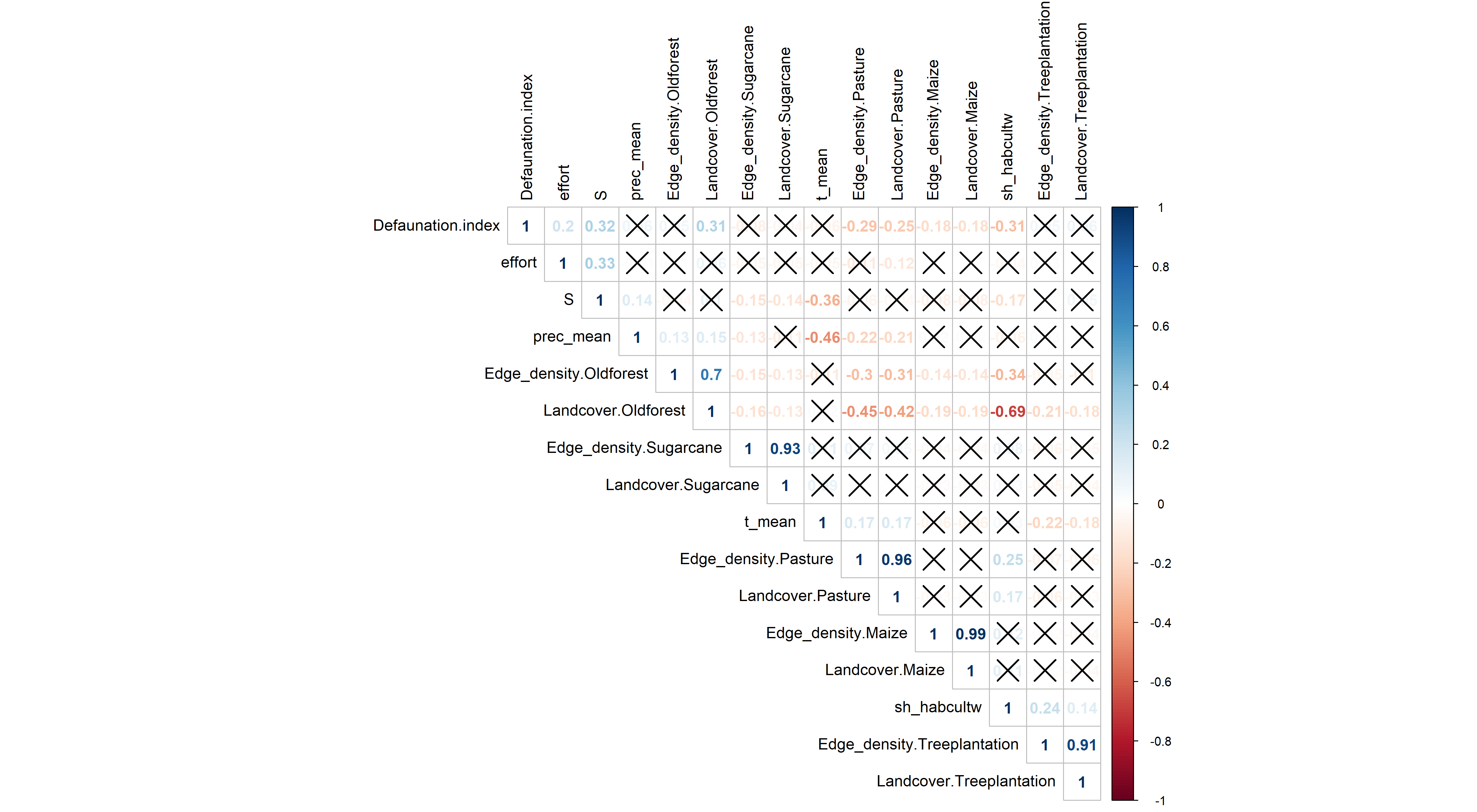

Supplement: S2 Fig — Crosses indicates non-significant correlations (p<0.05). We only used predictors with correlations lower than 0.4 in magnitude in the same model. S = small mammal local species richness, prec_mean = average rainfall, t_mean = average temperature. (TIF) [file pntd.0007655.s004.tif]

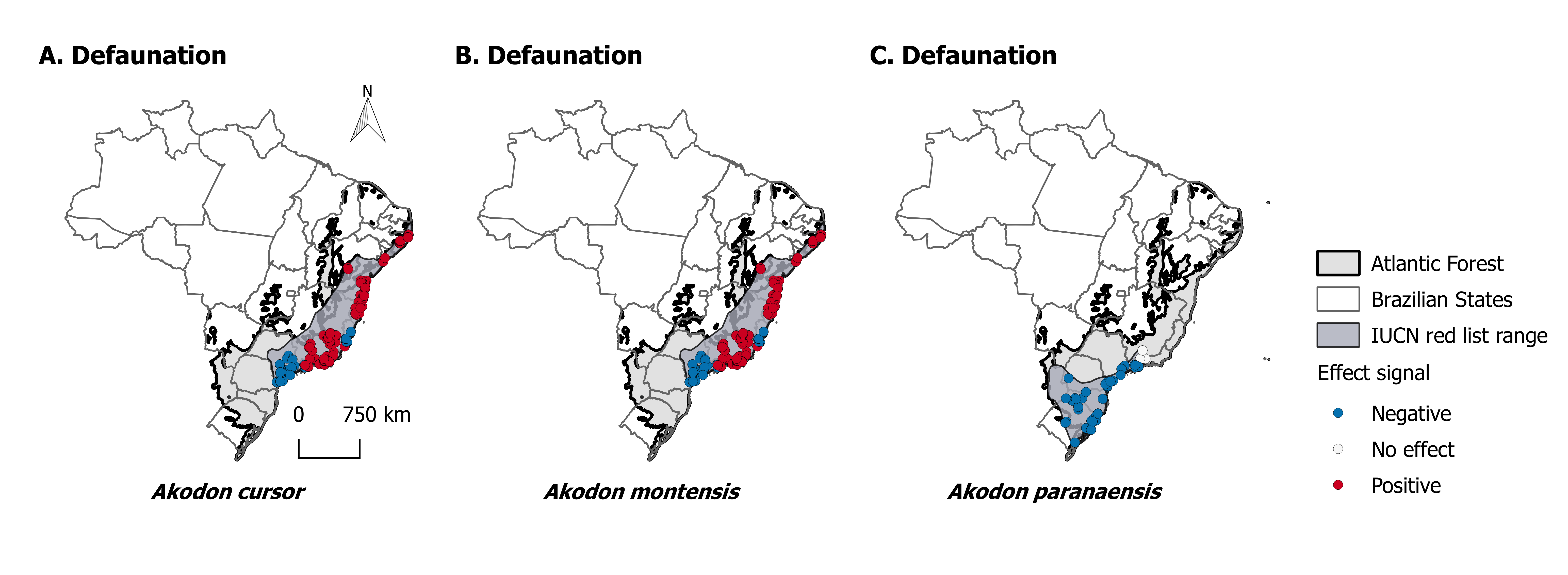

Supplement: S3 Fig — A. Akodon cursor, B. Akodon montensis and C. Akodon paranaensis proportions as a function of defaunation. Ranges were downloaded from IUCN red list website [44]. (TIF) [file pntd.0007655.s005.tif]
